# Study of Biomechanics of the Heart Valve Leaflet Apparatus Using Numerical Simulation Method

**DOI:** 10.17691/stm2022.14.2.01

**Published:** 2022-03-28

**Authors:** K.Yu. Klyshnikov, P.S. Onischenko, Е.А. Ovcharenko

**Affiliations:** Researcher, Laboratory of New Biomaterials, Department of Experimental Medicine; Research Institute for Complex Issues of Cardiovascular Diseases, 6 Sosnovy Blvd, Kemerovo, 650002, Russia; Junior Researcher, Laboratory of New Biomaterials, Department of Experimental Medicine; Research Institute for Complex Issues of Cardiovascular Diseases, 6 Sosnovy Blvd, Kemerovo, 650002, Russia; Head of the Laboratory of New Biomaterials, Department of Experimental Medicine; Research Institute for Complex Issues of Cardiovascular Diseases, 6 Sosnovy Blvd, Kemerovo, 650002, Russia

**Keywords:** aortic valve prosthesis, finite element method, hydrodynamic study of the prosthesis, leaflet valve apparatus, simulation of the prosthesis’ leaflet apparatus biomechanics, simulation of the bioprosthesis’ work

## Abstract

**The aim of the study** was to study the complex biomechanics of the aortic valve prosthesis and to analyze the effect of frame mobility on the stress-strain state and geometry of the valve leaflet apparatus using a numerical simulation method, which reproduces the qualitative and quantitative results of its bench tests.

**Materials and Methods:**

The object of the study was a commercial valve bioprosthesis UniLine (NeoCor, Russia), a three-dimensional mesh of which was obtained on the basis of computer microtomography with a subsequent analysis of its stress-strain state in the systole– diastole cycle by the finite element method in the Abaqus/CAE medium. The simulation was validated by comparing the results of numerical and bench simulation on the ViVitro Labs hydrodynamic system (ViVitro Labs Inc., Canada).

**Results:**

The method proposed in this study to simulate the mobility of commissural struts by including elastic connectors of adjustable stiffness in the calculation made it possible to reproduce the qualitative effects of the valve leaflet work observed in the bench experiment. The bioprosthetic orifice area in the systolic phase corresponded to the values obtained in the hydrodynamic system throughout the entire systole–diastole cycle. The analysis of the stress-strain state has shown the fundamental difference in the distribution of the von Mises stress fields depending on the numerical experiment design: the concentration of high amplitudes in the area of commissural struts and the central part of the free edge. However, quantitatively, the stress values reached the maximum of 0.850–0.907 MPa (0.141–0.156 MPa on average), which is below the ultimate strength of the biological material.

**Conclusion:**

The results of this study with the validation performed allowed us to conclude that adequate results of modeling the biomechanics of the heart valve leaflet bioprosthesis based on the finite element method can be achieved by using a high-resolution model with the imposition of elastic connectors in the area of commissural struts. Taking into account the mobility of the frame struts of the heart valve prosthesis is decisive in relation to the final geometry of the valve apparatus and can act as a negative factor in case of a highly elastic material of the valve apparatus. The simulation method presented can be used to optimize the leaflet apparatus geometry of heart valve prostheses from the standpoint of assessing the distribution of the stress-strain state.

## Introduction

In modern medical science, numerical methods of simulating the work of heart valve bioprostheses are being actively used at the stages of their design and optimization as the recognized tools of an in-depth engineering analysis [1-4]. The main motivation of *in silico* studies is to increase the lifetime of the prostheses [5, 6] functioning under the condition of a long-term load in the recipient organism and to improve their hemodynamic performance [1, 2, 7]. The literature shows a direct dependency of pathological mineralization and fatigue-induced deterioration of the leaflet apparatus on the stress and strain amplitudes in its material [8-11]. Therefore, distribution optimization of the given characteristics is able to increase the fatigue strength and the time of dysfunction development, which nowadays is about 10–15 years [12-14]. Besides, *in silico* simulation allows to reveal the potential for improving the hemodynamic characteristics of the prostheses (enlargement of the opening area, decrease of transprosthetic gradient, and regurgitation volume) since the work of diverse leaflet apparatus geometries may be calculated prior to the stage of natural prototyping [1, 7]. The results of optimization and understanding of the interconnection in the design– effect system underlie the creation of novel, more effective models of bioprostheses [15] and, in the long run, for achieving better results of cardiac valve surgical intervention from the standpoint of clinical parameters.

**Figure 1.**
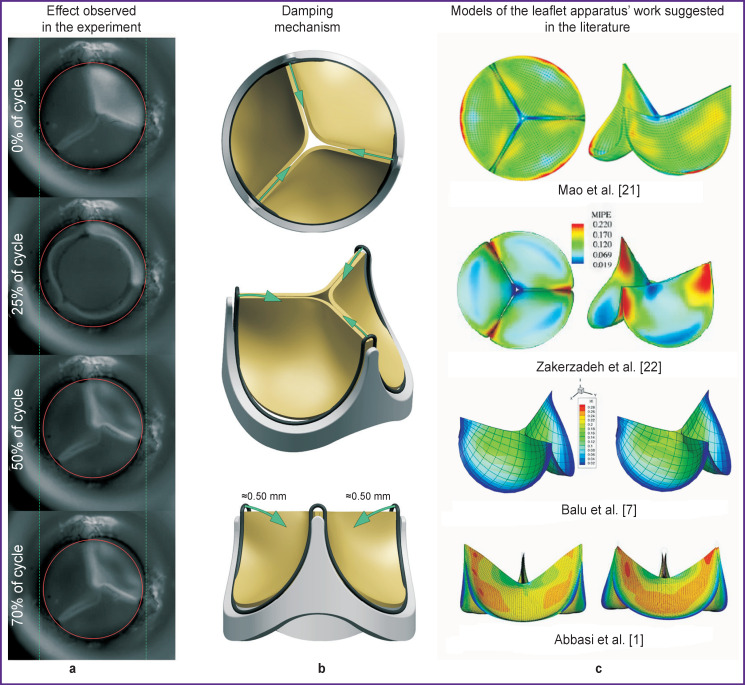
Hemodynamic effect appearing for valve bioprostheses with a semi-rigid supporting frame: (a) the change in the strut position and work of the supporting frame leaflet apparatus for hydrodynamic damping observed during systole–diastole cycle: a circle built across the three points of the frame changes its diameter; (b) visualization of the damping mechanism realized by the mobile struts of the semi-rigid supporting frame of the valve prosthesis; direction of the frame strut movement is demonstrated; (c) typical examples of simulating the leaflet apparatus work described in the literature

Numerical simulation requires indispensable simplification of the problem statement: some assumptions in the material behavior, reduction of the border conditions and geometry, whose application validity must be grounded qualitatively and quantitatively in comparison with a natural *in vitro* or *in vivo* experiment [16, 17]. A number of articles on the numerical simulation directed to the search for an optimal leaflet apparatus geometry of the cardiac valve prostheses reproduce the basic functional of its work [1, 2, 7, 18, 19], although fail to provide minute qualitative repetition of the intricate effects and show qualitative disagreement of the results with the experiment. The biomechanics of bioprosthetic valve components for hydrodynamic damping in the diastolic phase may be referred to such a complicated problem [20]. Such behavior is typical for the prostheses with a semi-rigid supporting frame which, when locking, is capable of deforming reversibly (elastically) in the area of the commissural struts and reducing the tensile load on the leaflet (Figure 1). However, the majority of the current *in silico* numerical calculations do not imply this possibility restricting completely the motion of the leaflet apparatus along the sewing margin.

The described experiments [1, 21-23] (Figure 1 (c)) distort the pattern of the leaflet apparatus behavior and consequently the quantitative results of the stress-strain state analysis obtained in this way may be incorrect. Therefore, **the aim of this study** was to modify the existing approaches to the numerical simulation of the work of the prosthetic valve leaflet apparatus in order to reproduce the specific performance of its biomechanics with validation of the calculation in the *in vitro* experiment.

## Materials and Methods

***The object of the study*** in the present work was a 23-mm UniLine aortic valve bioprosthesis (NeoCor, Russia). The leaflet apparatus work of this bioprosthesis is characterized by a damping effect due to the mobile semi-rigid supporting frame. The three-dimensional model of the clinical bioprosthetic specimen was redesigned using computed microtomography method on the OREL-MT system (Tomsk Polytechnic University, Russia): 880 slices of DICOM images with 2.54 μm resolution and 256 shades of gray. Next, using the tools of medical graphic data analysis, a spatial three-dimensional model was created consisting of the triangular facets forming a united polygonal body in the STL format. This model was imported into the NX CAD environment (Siemens, Germany) and redesigned into the solid model of the leaflet apparatus on the basis of the curved surface. Thereafter, a thickness of 0.5 mm was assigned to the surface: an average value for a commercial xenopericardium of the cattle used in fabrication of this bioprosthesis.

### Numerical simulation — convergence analysis

The solid models of the leaflets obtained were imported into the Abaqus/CAE engineering analysis medium (Abaqus Inc., USA) and their biomechanics was simulated numerically using the finite element method. For this purpose, a finite-element mesh was used for interpolation for each leaflet. The size of the finite element was identified in the preliminary convergence experiment during which the von Mises stress and maximal strain in the material in the open leaflet condition were assessed using a simplified tricuspid valve operation model. The most detailed final-element mesh with 0.1-mm element edge length and total number of elements of 371,775 served as a reference model. The edge size was changed in the following succession: 0.1, 0.2, 0.3, 0.4, 0.5 mm modeling similar working conditions of the prosthesis. The criterion of the optimal element size was a 5% deviation from the most accurate finite-element mesh with a 0.1-mm ridge length.

As a result, the obtained model may be described in the following way: the element type — a hexahedral element of the first order C3D8; 0.4-mm average edge length; total number of elements per tricuspid model — 7224; total number of nods — 11,376.

### Numerical simulation — leaflet biomechanics

The main *in silico* experiment on modeling the leaflet apparatus work was carried out using Abaqus/Explicit solver including explicit time integration for maximally physiological reproduction of the leaflet work. In the course of simulation, the leaflet operation was calculated during two systole–diastole cycles lasting for 0.857 s each, which corresponds to the heart rate of 70 bpm. The first cycle is supposed to serve for calculation stabilization, the second — for direct reception of the quantitative results.

The model of the material for the leaflet apparatus was selected based on the results mechanical testing applying uniaxial tension to the cattle xenopericardial patches (n=5) on the Zwick Z50 testing machine (Zwick/Roell, Germany). The obtained stress–strain curves were imported in the form of data for building a polynomial model of the second order — reduced polynomial. Contact interactions of the leaflets with each other were described pairwise based on the

**Figure 2.**
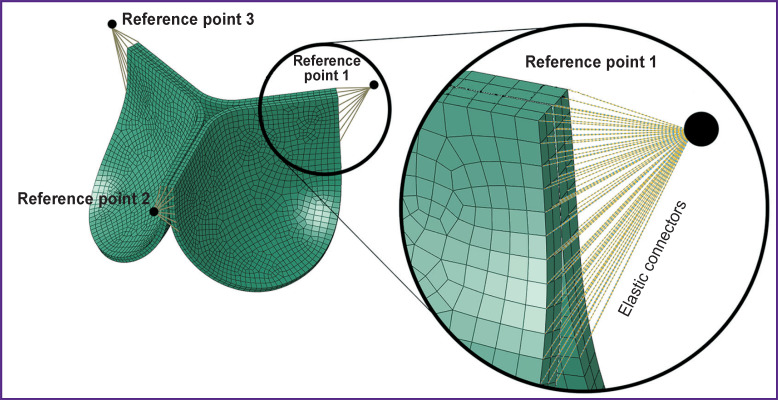
Specific aspects of the numerical experiment setup providing mobility of the bioprosthesis commissural strut for hydrodynamic damping: inclusion elastic connectors into calculations

Coulomb friction model: in the tangential direction — by a penalty method with a 0.2 coefficient of friction; in the normal one — with a 0.2 linear coefficient of stiffness. The pressure applied separately to the ventricular and aortic surfaces of the leaflet apparatus served as a model of the load imitating the action on the leaflet apparatus. Besides, the pressure amplitude reproduced the results of the natural test of this UniLine prosthesis obtained on the hydrodynamic system (see “Validation” section). Two cases of the leaflet apparatus operation were simulated with:

- mobile commissural struts — to imitate the mobility of the supporting frame struts damping a hydrodynamic shock, elastic connectors were employed to connect reference fixed points and separate nods of the leaflet apparatus in the area of commissures (Figure 2). These connectors provide controlled mobility of the leaflet apparatus in the radial direction along the ρ and z axes (for the cylindrical coordinate system) owing to their radial-thrust type;
- immobile commissural struts — a more “traditional” experiment setup was used as a comparison option by analogy with those described in the literature — without the possibility of commissural strut motion [1, 21, 22].

The stress-strain state parameters, i.e. von Mises stress amplitude and logarithmic strain, as well as specific localization in the leaflet areas, were used to compare the two simulation cases.

### Validation

**Figure 3.**
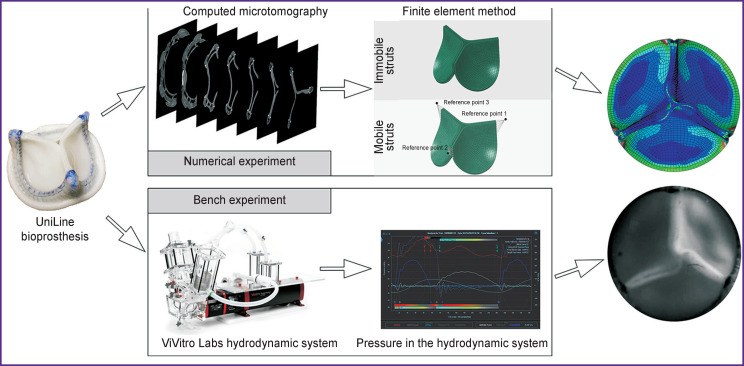
General concept of studying leaflet apparatus’ biomechanics: numerical simulation of the aortic valve bioprosthesis’ work and bench tests using hydrodynamic system with subsequent comparison of the qualitative-quantitative results

The key stage determining the consistency of the proposed simulation approach is the stage of validation: qualitative and quantitative comparison of the prosthesis biomechanics parameters with the results of the bench testing (Figure 3). To validate the calculation, a digitized specimen of the UniLine prosthesis was tested on the ViVitro Labs testing machine (ViVitro Labs Inc., Canada) under the imitated normal functioning of the aortal valve. The stroke volume was 5 L/min, heart rate — 70 bpm, aortic pressure — 120/80 mm Hg. The bioprosthesis operation was recorded using a Fastvideo-250 high-speed video camera (Fastvideo, Russia) at 250 fps. The following criteria were used to compare the results of the numerical simulation and bench experiment:

- quantitatively — the area of the leaflet apparatus opening defined as an area of the passage orifice on each frame of the slow motion video on the hydrodynamic system and each frame in the process of simulation. Statistical differences were assessed by a pairwise comparison of the independent samples using the Mann–Whitney test. Differences were considered significant at p<0.05;
- qualitatively — the behavior of the leaflet apparatus and the effects occurring on the key frames of leaflet motions: beginning of opening, maximal opening, locking of the leaflet apparatus, maximal closure of the leaflets.

## Results

**Figure 4.**
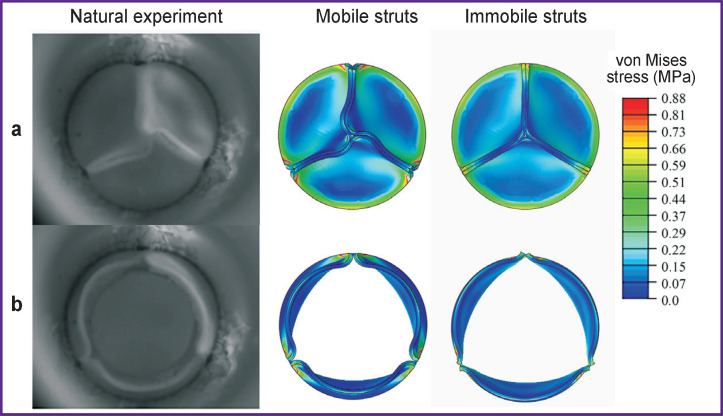
Qualitative comparison of the two variants of leaflet apparatus’ work simulation with the bench tests in two positions: (a) peak of the closed state; (b) peak of the open state

In the process of numerical simulation, it has been established that inclusion of the elastic connectors, providing mobility of the commissural struts of the leaflet apparatus, into calculations changes essentially the model behavior and quantitative results. Such *in silico* experiment successfully reproduces leaflet closure and opening corresponding to the bench validation. On the contrary, in case of prevention of the commissural strut movements, the biomechanics in the numerical model differs radically from the results of hydrodynamic testing. This result is well demonstrated graphically (Figure 4), especially for the phase of the locked leaflet apparatus. Overall, the completely open state of the leaflet work did not differ qualitatively.

**Figure 5.**
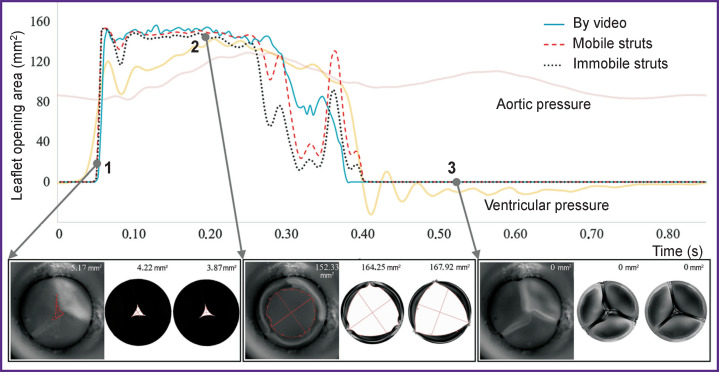
Quantitative analysis of changes in the orifice area of the leaflet apparatus in the process of bench tests for validation and simulation options Visualizations of different stages of cardiac cycle are presented: start of leaflet opening (*1*), peak of opening (*2*), fully closed state (*3*)

The frame-by-frame analysis of the opening area for the two simulation cases has demonstrated insignificant disagreement with the validation results (Figure 5). Statistically significant differences in the opening areas were not detected.

The quantitative characteristics of the stress-strain state also differed insignificantly. Thus, for the case of mobile commissural struts, the stress amplitude in the biological material was comparable with the variant of fully immobile struts. In the first case, the stress maximum amounted to 0.850 МPа, in the second — to 0.907 MPa. The average von Mises stress was 0.141±0.094 and 0.156±0.075 MPa, respectively. The value of the logarithmic strain in simulated variants also did not differ: for the mobile struts, it was 0.27 m/m, for the immobile — 0.24 m/m. The average value was 0.078±0.0001 m/m and 0.082±0.0003 m/m, respectively.

**Figure 6.**
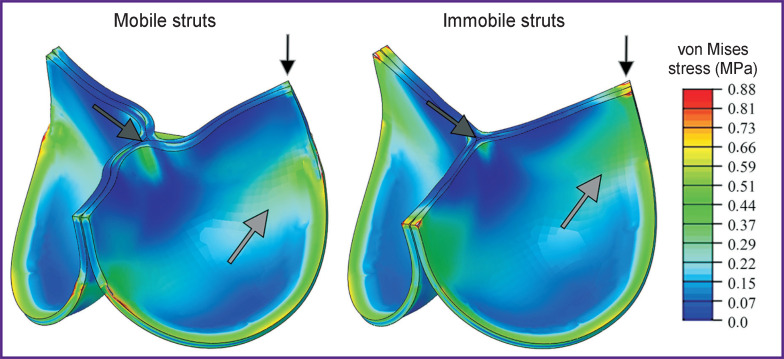
Diagrams of von Mises stress distribution for the two studied cases of behavior of prosthetic valve leaflet apparatus Regions of the leaflet with different distribution of the von Mises stress fields are indicated pairwise by arrows

Qualitative characteristics, distribution diagrams for these values, had differences primarily due to the damping effect and folded central area of the leaflet free edge. When elastic connectors were used, a more uniform stress distribution across the leaflet volume was observed while for the immobile struts, the main stress fields were concentrated in the area of commissures (Figure 6). Thus, as the result of the immobile strut calculations, areas experiencing significant loads, mainly tension, were observed. On the other hand, the effect of leaflet twisting in the closed state for the case of the mobile commissures formed flexures in the central part of the free edge, resulting in the stress growth up to 0.52 MPa in this area. In the alternative case, the flexure was not formed and the stress reached 0.35–0.40 MPa.

## Discussion

The results of numerical simulation depend largely on its settings and the degree of granularity of the object behavior being reproduced [23]. Regarding computer analysis of the complicated multicomponent system operation, i.e. the biological heart valve prostheses in this case, this factor may cause serious distortion of the results on the basis of which conclusion on safety and effectiveness of the medical device is made. This is why the question of numerical experiment validation for these devices is a pressing issue. Unfortunately, researchers do not always pay due attention to the comparison of the proposed numerical calculation of bioprosthetic biomechanics with natural bench tests, which leads to a paradoxical situation. On the one hand, one can find in the literature the description of a sufficient number of high-level approaches to an *in silico* study of the leaflet apparatus of the heart valve bioprostheses, and many of them are modeling, among other things, sophisticated multidisciplinary interactions (multiphysics) as well. The most explicit of these studies reproduce the liquid–solid body processes with a complex description of materials [24, 25], detailed selection of boundary conditions, and imitation of a multicomponent liquid, such as blood [5, 26], but their results in the majority of cases are not compared with the results of the bench tests or do not deliberately reproduce important effects observed in the *in vitro* experiment in the modeling process [27, 28]. At the same time, the researchers in their works make the conclusions on the interrelation between the leaflet geometry and hydrodynamics [2, 29], the leaflet form and cooptation zone size [7], i.e. the properties directly associated with the risks and effectiveness of bioprosthesis application. How safe is it to rely on these conclusions obtained without proper validation when designing a medical device of the highest risk class (heart valve bioprosthesis) remains a serious question.

**Figure 7.**
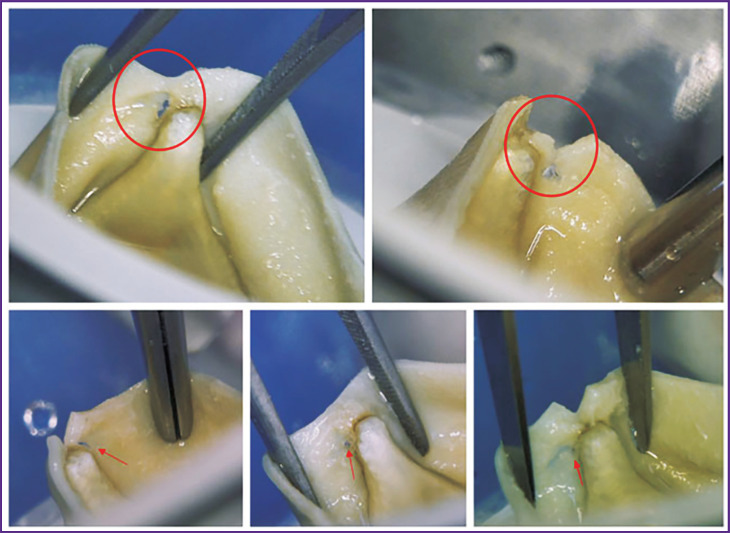
Visualization of leaflet ruptures in the areas of commissural struts of Trifecta prostheses (Abbott, USA) for which a similar effect was observed in five of nine cases after 500 million cycles during cycle durability tests [30]

The problem is considerably aggravated by the occurrence in the *in vitro* experiment atypical events in the bioprosthesis work characteristic, for example, for our object under consideration: hydrodynamic damping by the supporting frame is due to the mobility of its struts. This damping concept is grounded [17] and represents an attempt to solve the problem of tearing the bioprosthetic leaflet off the commissural strut exposed to long-term loads for the case of a stiff immobile frame (Figure 7) [30]. However, practically all models presented in the literature do not have the capacity and tools to imitate this specific behavior despite the in-depth elaboration of other aspects: nonlinear materials, complex loads, physics of liquids.

Nevertheless, it should be noted that two *in silico* investigations, which considered a damping effect in the context of modeling the durability of the leaflet apparatus, were described in the literature [6, 31]. In these works, different variants of commissural strut deviations in a Carpentier-Edwards PERIMOUNT bioprosthesis (USA) were imitated, however, no validation of this behavior was performed. It is noteworthy that the authors of these articles come to a similar conclusion that is observed in our study: the mobility of the prosthesis struts eliminates the fatigue leaflet damage in the area of commissures but increases damage to the free ridge. Evidently, it is from this position, i.e. reduction of stress amplitudes in the area of a free ridge, that further optimization of the studied UniLine prosthesis should be done. Despite a successful history of applying this prosthesis in clinical practice [32-34], its leaflet apparatus has a potential for improvement and the proposed setup of the *in silico* experiment may become a valuable tool for the assessment of the effectiveness of these improvements by numerical analysis.

## Conclusion

Here, we present the approach to simulating the work of the leaflet apparatus of the heart valve prosthesis taking into account the mobility of the commissural struts and hydrodynamic damping. The approach was released by the introduction in the model additional elastic connectors of regulated stiffness, which provided restricted controlled motion of the leaflet commissures. Application of this approach has been shown to make the distribution of the stress and strain fields in the leaflet apparatus material considerably more accurate. Besides, it reproduces quantitative and qualitative results of validation under the bench test conditions imitating the work of the valve prosthesis, i.e. has natural substantiation.

The results of the UniLine prosthesis biomechanics modeling have demonstrated that mobility of the frame struts of the heart valve prosthesis is a crucial aspect in assessing the effectiveness of the leaflet apparatus geometry and may be a negative factor in case of a highly elastic leaflet material.

The presented modeling method can be used to optimize the geometry of the leaflet apparatus of heart valve prostheses fabricated from both xenogenic canned biomaterials and synthetic polymers from the standpoint of assessing the distribution of the stress-strain state.
